# The promise – and pitfalls – of smoke-free policy adoption

**DOI:** 10.1186/s13584-019-0313-9

**Published:** 2019-05-03

**Authors:** Vaughan W. Rees

**Affiliations:** Department of Social and Behavioral Sciences, Center for Global Tobacco Control, Harvard T.H. Chan School of Public Health, Kresge Building, 6th Floor, 677 Huntington Ave, Boston, MA 02115 USA

**Keywords:** Environmental tobacco smoke, Secondhand smoke, Smoke-free policy, Tobacco control, Cotinine, Israel

## Abstract

Environmental Tobacco Smoke (ETS) is a major, preventable cause of morbidity and mortality, disproportionately impacting vulnerable populations. Policy measures, guided by the WHO’s Framework Convention of Tobacco Control, have focused on the broad adoption of smoke-free laws. While smoke-free policies are effective in reducing ETS exposure, limited policy dissemination and suboptimal implementation strategies have limited their impact.

New research reported by Berman and colleagues in this journal brings these issues into sharper focus. Substantial advances in tobacco control policy have been achieved in Israel, including widening of smoke-free laws, since the passing of a Knesset bill in 2012. However, Berman and co-authors present found no reduction in ETS exposure in a nationally representative sample of non-smoking Israeli adults in 2016 compared with an earlier benchmark measured in 2011. In line with research from international settings, they found that ETS exposure was higher among a traditionally vulnerable subpopulation. The findings serve to remind us that the mere adoption of a policy will not translate into meaningful public health impact without applying best practice implementation strategies. Above all, this work emphasizes the continual need for new research to improve existing policies and inform new policy approaches in pursuit of an end to the harm arising from the global tobacco epidemic.

The global tobacco epidemic continues to exact a massive toll on human health. While the harms caused directly by smoking are now widely recognized, the harms associated with exposure to secondhand smoke are often overlooked. Secondhand smoke, also referred to as environmental tobacco smoke (ETS), contains some 7000 chemicals, of which some 250 are known to be toxic and 69 carcinogenic [1, 2]. ETS exposure causes serious adverse health outcomes, including respiratory illness, heart disease and lung cancer, and is attributed with approximately 800 deaths per year in Israel [3] and 34,000 deaths per year in the United States [4]. Children have special vulnerability to ETS exposure, which increases risk of acute respiratory infections, ear infections, severity of asthma symptoms, and sudden infant death syndrome (SIDS) [1]. Marked disparities in ETS exposure have been noted: in the U.S. for example, exposure remains highest among young children aged 8–11, those of low SES, those living in rental housing and non-Hispanic blacks [5]. These disparities underscore the need for rigorous health policy measures that protect not only the general population from ETS exposure, but also reach the known vulnerable populations who experience a disproportionate burden of harm.

With the guidance of the WHO’s Framework Convention on Tobacco Control — the first global public health treaty — smoke-free laws have been adopted in many national and subnational jurisdictions. While smoke-free laws were initially conceived as a strategy to protect workers in occupational settings such as hospitality venues, they have steadily expanded to encompass a diverse range of public settings, including airports, health and educational facilities, and many business and recreational facilities [6]. As a result of broad dissemination, smoke-free laws are now attributed with benefits beyond simply preventing ETS exposure. Research has shown that smoke-free laws encourage cessation among smokers, and, because smoke-free laws have made smoking less socially acceptable, may also prevent young people from initiating smoking [7]. These benefits ensure that smoke-free laws maintain strong public support in jurisdictions where they have been introduced [8, 9]. Nevertheless, there are concerns that not all smoke-free laws conform to the FCTC-recommended best practice recommendation for comprehensive coverage: for example, policies that allow designated smoker areas, exceptions based on building design or outdoor seating, or other loopholes do not provide full protection from ETS exposure [10]. And despite their proliferation and popular support, just 20% of the world’s population enjoys the protection of a comprehensive national smoke-free law [11].

New research reported by Berman and colleagues [12] underscores concerns about the impact of smoke-free laws in Israel. In a cross-sectional analysis of *N* = 194 adults who participated in the 2015–16 National Health and Nutrition Survey (RAV MABAT), they found that 63% of non-smoking Israelis were exposed to ETS. This finding is particularly disappointing in light of evidence that there has been no reduction in the proportion of non-smokers exposed to ETS or the concentration of cotinine, an ETS biomarker, since 2011, when these investigators analyzed the same outcomes in an earlier Israeli cohort [13]. Of particular note is the fact that smoke-free policies were dramatically expanded in Israel in 2012, bringing enhanced ETS protection to medical facility entrances, train stations, outdoor swimming pools and government offices. Smoking bans in Israel were further extended to sports stadiums in 2014 and schools in 2016. With bans covering a wider range of settings, we should expect to see fewer non-smokers exposed to ETS and lower concentrations of the cotinine biomarker among those who are exposed.

These perplexing findings raise the question: have hard fought tobacco policy gains, delivered via Knesset vote, produced little benefit to the health of the public? Experience has taught us that political will is predicated on public support, which is more likely to arise when policies are perceived as beneficial. Could these findings prove to be a setback in Israel’s ongoing policy efforts to curb the tobacco epidemic?

While Berman and colleagues provide only limited insights on the possible reasons why ETS exposure has not declined, they nonetheless point to a forward path. The authors appeal for increased enforcement of Israel’s far-reaching laws to support greater policy impact. PoorlyP enforced smoke-free policies are less likely to yield the benefit of ETS exposure reduction, and enforcement of existing policies requires constant vigilance [14]. Smoking bans are also more effective when accompanied by health communication campaigns that inform the public about the harms of ETS exposure and provide a rationale for bans. Berman and colleagues thus call for further efforts to educate the public on the reasons and benefits of smoking bans. Another important recommendation is the author’s assertion that current smoke-free laws in Israel have not been extended far enough. They argue that more bans on smoking should be extended to outdoor public places, including playgrounds and sports facilities. Innovative policy approaches in global settings have seen the adoption of smoking bans in public parks, beaches, and even public streets. These policies serve to “denormalize” smoking, leading to lowered smoking rates which may in turn lower exposure among non-smokers. Smoke-free policies must reach underserved populations, where disparities in ETS exposure continue to exist. Data from the current study show that ETS exposure is higher among Arab non-smokers compared with Jewish, and that exposure that occurs in the home is associated with higher cotinine concentrations. Policies that ban smoking in low income residential settings, such as multi-unit dwellings and, in the U.S., in federally-subsidized public housing may help to protect those of low socio-economic status [15–17]. Other investigators have emphasized that mere policy *adoption* is not sufficient without effective policy *implementation,* encompassing better public communication and engagement, strategies to assist compliance, and rigorous enforcement [18]. Effective implementation strategies not only can increase policy impact, but also enhance public trust, paving the way for future policy initiatives.

## Conclusions

Israel stands at a critical point in tobacco control policy. Difficult battles have yielded a raft of valuable policy initiatives, including the smoke-free laws recommended under the FCTC. However, Berman and colleagues show us that there is much progress still to be made. As those working in tobacco control research and policy consider the tobacco “end game” — the steps necessary to finally rid the globe of the tobacco epidemic — lessons from Israel emphasize that we must improve existing policies, develop new policy strategies, and improve policy implementation, if we are to overcome this pervasive public health problem.
